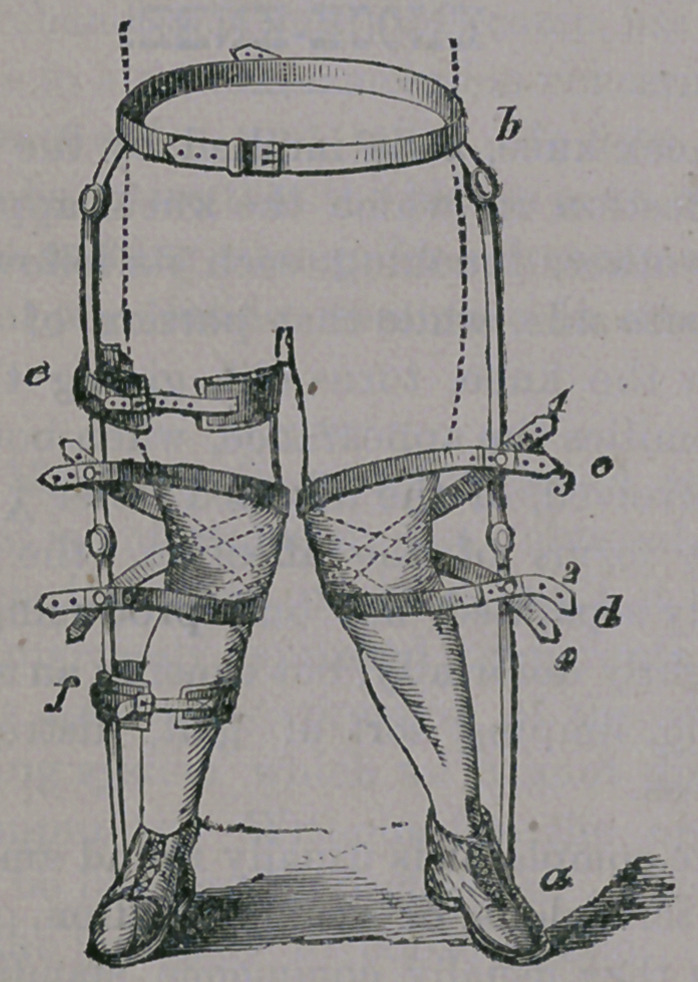# Knock-Knee

**Published:** 1874-10

**Authors:** 


### KNOCK-KNEE.

Knock-knee, as is implied by the name, is an affection in which the knees approximate each other, touching each its fellow on the opposite side, while that portion of the limb below the knee, turns out, giving the lower extremities the appearance, when both knees are involved, of the inverted letter ⅄. In the worst forms of the affection, the feet are widely separated, not only producing a very unsightly deformity, but causing an awkward, rolling, limping sort of gait, distressing to witness.

The complaint is usually found among enemic, scrofulous, or weakly children, of the age when they usually commence standing upon the mother's lap, until they have reached sixteen, when the deformity reaches its utmost limit. The affection seems to be most prevalent among the boys, but just why, to us does not seem entirely clear. The deformity depends upon a relaxed, enfeebled or paralyzed condition of the *internal lateral ligament*, which allows the muscles upon the opposite side to drag the heads of the bones of the leg outward. In children of five years and upward, it is often caused by being employed at some work requiring them to stand, or in lifting heavy parcels. We have seen it caused in young men whose occupation required them to use the limb in treading a lathe or bellows. In such cases usually but one limb is affected, the one used in the labor referred to. Book-keepers, who perform their duties while standing at the desk, all day long, sometimes are victims to the deformity. Infants, born with "rickets" favor a development toward this trouble. But, from whatever cause the deformity may originate, it demands immediate attention. It is a serious error to imagine that the child will "out-grow it," or that the deformity will become spontaneously rectified. This never happens,—and the longer suitable treatment is neglected, the more difficult it is of cure. In the earlier stages, its treatment presents but little difficulty, the limbs being readily restored to their proper position. In the case of an infant, a well-padded splint of bookbinders' board, placed to the outside of the limb and snugly secured by a well-applied roller bandage, will be all the treatment necessary. In older children, an appliance as here illustrated,—

will be required. It consists of a steel waist band, to which are secured light steel braces, with suitable joints, and secured to stout shoes. Pads and straps are then placed about the limbs at such points as the surgeon deems necessary to overcome the contraction of the opposing muscles, and are secured to the braces as illustrated. In still older subjects, it often becomes necessary to sever, subcutaneously, the tendon of the external hamstring, before the limbs can be fully restored. Indeed, in most of the severe cases with which the surgeon has to deal, more or less cutting of the tendons involved in the difficulty, become necessary to form a speedy cure. The subcutaneous divisions of the tendons and muscles is such a painless and harmless procedure, under the skillful hand of an experienced surgeon, that this course is almost invariably adopted as the most expeditious and the least painless of all the methods employed. After the operation, a light apparatus is worn for a few months, under the clothing, when the cure becomes complete and satisfactory.

## Figures and Tables

**Figure f1:**